# ProSAAS overexpression arrests progression of motor deficits in a rat alpha-synuclein model of Parkinson’s disease

**DOI:** 10.1186/s40035-026-00593-x

**Published:** 2026-10-16

**Authors:** Zhan Shu, Hoa A. Lam, Elizabeth J. Videlock, Iris Lindberg, Nigel T. Maidment

**Affiliations:** 1 Department of Psychiatry and Biobehavioral Sciences, Hatos Center for Neuropharmacology, Semel Institute for Neuroscience and Human Behavior, UCLA School of Medicine, Los Angeles, CA USA; 2 Division of Digestive Diseases, Department of Medicine, UCLA School of Medicine, Los Angeles, CA USA; 3 Department of Neurobiology, University of Maryland School of Medicine, Baltimore, MD USA

Current medical treatments for Parkinson’s disease (PD) address symptoms but do not impact disease progression. Protein chaperones offer a promising means of restoring proteostasis in PD and other proteinopathies. ProSAAS, a neuroendocrine peptide precursor protein [1] associated with Lewy bodies [2], was recently identified as a potential biomarker in PD [3, 4]. ProSAAS harbors a central unprocessed core chaperone sequence that prevents alpha-synuclein (α-Syn) fibrillation in vitro [2], and proSAAS overexpression prevents induction of α-Syn toxicity in vivo when initiated simultaneously with α-Syn insult [5]. However, at the time of PD diagnosis, dopamine (DA) terminal loss is already profound. It is therefore critical that preclinical evaluations of agents with therapeutic potential demonstrate efficacy when initiated after onset of significant pathology. Here, we provide evidence of such efficacy in an animal model when nigral proSAAS overexpression is initiated several weeks after the onset of motor deficits.

Male rats received a unilateral injection of an AAV expressing human α-Syn (hα-Syn) into the substantia nigra (SNc) followed 4 or 6 weeks later (Fig. 1a, left and right panels, respectively) by injection of lentivirus expressing either proSAAS or GFP into the same region (Additional file 1: Supplementary Methods). AAV-hα-Syn expression (confirmed by immunocytochemistry, Fig. S1) induced progressive sensorimotor (forelimb bracing and placement tests) and spontaneous motor (cylinder test) asymmetry (Fig. 1b). Strikingly, in the initial 4-week delay experiment, progression of spontaneous motor asymmetry was arrested, and sensorimotor asymmetry was completely reversed, by lenti-proSAAS (Fig. 1b, left). Post-mortem analyses conducted in a randomly selected subset of animals (Additional file 1: Supplementary Methods) demonstrated that proSAAS over-expression (confirmed by immunocytochemistry, Fig. S2) attenuated the asymmetry in TH terminal density throughout the striatum (Fig. 1c, left) and prevented the stereologically assessed reduction in nigral TH^+^ neuron counts relative to the contralateral structure, observed in the lenti-GFP treatment controls (Fig. 1d, left), at 8 weeks post-AAV-hα-Syn insult (Fig. S3a shows Nissl^+^/TH^−^ data).

**Fig. 1:**
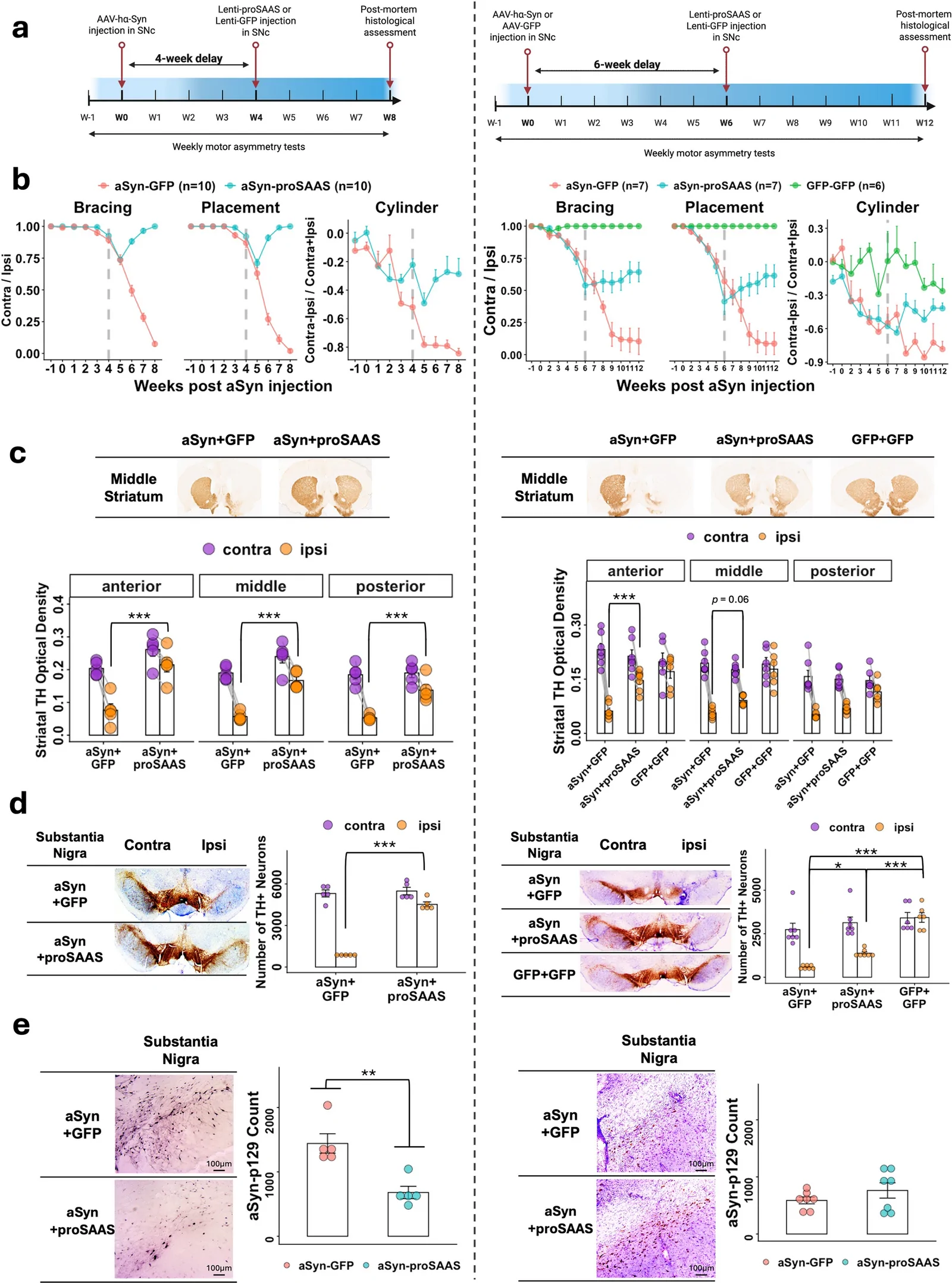
Four-week (left panels) and six-week (right panels) delayed proSAAS overexpression arrests progression. **a** Experimental time-course. **b** Progression of sensorimotor asymmetry (bracing and placement tests) was reversed or arrested by nigral proSAAS overexpression depending on the point of intervention (grey dashed line). Spontaneous motor asymmetry (cylinder test) was arrested in both experiments. Two-way mixed ANOVA of aligned rank-transformed data, followed by Tukey-corrected multiple comparisons where appropriate, confirmed significant differences between the treatment groups (*P* < 0.05–0.001, *n* = 10/group and 6–7/group for 4-week and 6-week delay experiments, respectively. No animals were removed from any analyses). **c** Asymmetry in TH terminal density throughout the striatum was measured in a randomly selected subset of 5 animals/group that were also subjected to nigral stereology. TH terminal density asymmetry was significantly attenuated by proSAAS in the 4-week delay experiment (left panels), confirmed by 3-way mixed ANOVA followed by pairwise comparisons (***P < 0.001, *n* = 5/group). Similar effects were observed in the 6-week delay experiment (right panels) but statistical significance was restricted to the anterior striatum (***P < 0.001, *n* = 6–7/group). **d** ProSAAS treatment prevented the reduction in nigral TH^+^ neurons induced by hα-Syn expression, measured stereologically in the subset of 5 animals per group at 8 weeks in the 4-week delay experiment (left panels); 2-way mixed ANOVA followed by post-hoc pairwise comparisons; ***P<0.001, *n* = 5/group. A significant but less pronounced proSAAS treatment effect was observed in the 6-week delay experiment (right panels); ***P < 0.001; *P < 0.05, *n* = 6–7/group. **e** Fewer p129-α-Syn immunoreactive cells were apparent on the side of injection in animals injected with lenti-pro-SAAS vs lenti-GFP, measured stereologically in the 4-week delay experiment (left panels); Students’ *t*-test, **P < 0.01, *n* = 5/group. No such difference was apparent in the 6-week delay experiment (right panels); *P* = 0.26, *n* = 7/group. Mean ± SEM in all figures

The complete protection of TH^+^ cells in proSAAS-treated animals at 8 weeks indicates a lack of significant DA cell death at the time of proSAAS virus delivery; the motor asymmetry evident at that time point resulted instead from TH downregulation and associated DA neuronal dysfunction, the progression of which was halted by proSAAS overexpression. To determine if proSAAS overexpression would be similarly effective when initiated later in the progression of pathology, after more extensive damage, a follow-up experiment employed a 6-week delay in lenti-proSAAS delivery (Fig. 1a, right). As expected, asymmetry was more profound at the point of proSAAS intervention (Fig. 1b, right). Nevertheless, proSAAS overexpression again arrested progression of spontaneous motor asymmetry and similarly halted further progression of sensorimotor asymmetry over the subsequent 6-week period. In this experiment, an additional control group received AAV-GFP followed 6 weeks later by GFP-encoding lentivirus. Significant loss of striatal TH terminal density was evident at 12 weeks in hα-Syn+GFP treatment controls relative to GFP+GFP controls, which was attenuated in the proSAAS-treated animals, most effectively in the anterior striatum (Fig. 1c, right). A considerable reduction in the number of TH^+^ cells was apparent in the SNc of hα-Syn+GFP animals. This reduction, while still profound, was significantly attenuated in proSAAS-treated rats (Fig. 1d, right) (Fig. S3b shows Nissl^+^/TH^−^ data).

We next sought evidence in support of a link between the observed protective effects of proSAAS and its previously reported anti-α-Syn fibrillation properties [2]. Phosphorylation of α-Syn at serine129 is a key post-translational modification associated with aggregated forms of the protein. Immunoreactivity for p129-αSyn is routinely used to infer the presence of such aggregates, although it likely also identifies pre-aggregated, but potentially more toxic, soluble oligomers [6]. Considerable numbers of p129-α-Syn-labelled cells were evident in the SNc of hα-Syn+GFP treatment controls on the injected side at 8 weeks in the 4-week delay experiment (Fig. 1e, left). Rats injected with lenti-proSAAS, despite having considerably more TH^+^ neurons (Fig. 1d), had significantly fewer p129-α-Syn-labelled cells (Fig. 1e, left). Notably, the number of cells exhibiting p129-α-Syn staining at 12 weeks in the 6-week delay experiment was similar between the two groups (Fig. 1e, right), but again, there were approximately 3-fold more remaining TH-positive neurons in the proSAAS-treated animals (Fig. 1d, right). This suggests that a lower proportion of surviving dopaminergic neurons exhibited pathological α-Syn in these treated animals. While not definitive, these data lend support to an α-Syn proteostatic mechanism of action.

The fact that sensorimotor dysfunction was reversed by proSAAS overexpression when initiated at 4 weeks, rather than its progression merely arrested, suggests that proSAAS may not only have limited the intercellular spread of pathological forms of α-Syn, as we previously reported [5], but also exerted a restorative function within impacted cells. Again, the parsimonious explanation, based on our previous data [2], is that proSAAS facilitated α-Syn proteostasis, likely following secretion and endosomal reuptake, promoting appropriate folding, targeting and degradation of overexpressed α-Syn. To investigate if such proteostasis includes promotion of clearance of already formed aggregates, we performed a follow-up experiment in which animals were injected with AAV-hα-Syn alone and euthanized 2–8 weeks later, to determine the extent of p129-α-Syn labeling at the point of proSAAS intervention. Data showed that p129-α-Syn labeling manifested as early as 2 weeks (Fig. S4a), concomitant with asymmetry in nigral TH^+^ cell counts (Fig. S4b) and striatal TH terminal density (Fig. S4c). While the number of p129-α-Syn-immunoreactive cells at 4 weeks in this experiment (Fig. S4a) was higher than that observed at 8 weeks in the proSAAS-treated animals in the 4-week delay experiment (Fig. 1a, left), a similar decline between 4 and 8 weeks was evident here, in the absence of proSAAS treatment (Fig. S4a). It is therefore not possible to conclude that proSAAS cleared already formed aggregates. Indeed, our previous in vitro data showed that proSAAS can prevent α-Syn fibrillation but cannot disaggregate already aggregated protein [2]. This may not prove important in potential therapeutic developments based on this approach given the growing consensus that Lewy bodies are not the toxic entity in PD but rather reflect the neuron’s attempt at mitigation of proteostatic dysregulation, and that soluble oligomers are the toxic species of concern [7].

Notably, the number of p129-α-Syn-labelled cells at 8 weeks in this follow-up study (Fig. S4a) was considerably lower than such counts in the hα-Syn+GFP group at 8 weeks in the 4-week delay experiment (Fig. 1e, left), while the reverse was true of TH^+^ cell counts, which trended slightly higher in the follow-up experiment at this timepoint (Fig. S4b vs Fig. 1d, left). This suggested that the ‘second hit’ of a lenti-GFP injection may have impacted the ability of cells to manage pathological α-Syn. AAV and lentivirus injections are proinflammatory and there is a considerable literature supporting a role for inflammatory processes in PD [8]. To preliminarily investigate whether inflammation may be an important component of this dual-virus model, we conducted a semi-quantitative immunocytochemical analysis of the SNc with the microglial marker, Iba-1, comparing the AAV-hα-Syn alone group at 6–8 weeks with staining at 8 weeks in animals injected with AAV-hα-Syn plus lenti-GFP from the 4-week delay experiment. While there was evidence of an inflammatory response to injection in both groups, there was no difference between the groups (Fig. S5a, b), suggesting that neither lentivirus itself nor GFP added to the inflammatory insult of the AAV-hα-Syn injection. The possibility that microglial engulfment of extracellular α-Syn accounted for the elevated p129-α-Syn-positive cells in the lenti-GFP-injected animals was also investigated but little colocalization of the two markers was evident (Fig. S6). An alternative explanation is that the additional proteostatic load imparted by GFP expression compounded that of the α-Syn insult, resulting in more accumulation of pathological forms of α-Syn.

Finally, the inflammatory response trended lower in lenti-proSAAS-treated animals (Fig. S5b). To investigate further whether this reflected an activity of proSAAS against a pro-inflammatory effect of the AAV vector itself or to the hα-Syn product, we conducted a similar analysis on tissues from the 6-week-delay experiment, which included an AAV-GFP + lenti-GFP control group. In this case, 12 weeks after the initial AAV-hα-Syn insult, inflammation was profoundly lower in the AAV-hα-Syn/lenti-proSAAS group compared to the AAV-hα-Syn/lenti-GFP group. However, a variable level of inflammatory response was also apparent in animals injected with AAV-GFP/lenti-GFP (Fig. S5c). Therefore, the anti-inflammatory effect of proSAAS may not be specific to inflammation induced by hα-Syn overexpression, but may nevertheless contribute to its therapeutic effect in the model by virtue of reducing the inflammatory response to virus injection. Interestingly, proSAAS is known to be terminally processed into several small bioactive peptides, in addition to the larger unprocessed central core chaperone sequence, LARALL [1]. These peptides include BigLEN, a ligand for the G-protein-coupled receptor, GPR171 [1], activation of which suppresses immune cell function [9]. While purely speculative at this point, increased availability of such peptides following proSAAS overexpression could plausibly attenuate any contribution of immune activation in the model and is worthy of further investigation.

It is important to recognize several limitations of this study. First, while the sample sizes employed are the norm for such initial preclinical investigations, extending such studies to larger numbers of animals, including females, and to other PD models, such as the α-Syn pre-formed fibril model, will be important going forward. Also, biochemical measures of oligomer/fibril burden and/or demonstration of proteinase-K resistance of p129-α-Syn immunoreactivity will be necessary to discern if proSAAS is impacting soluble versus aggregated forms of the protein. Finally, a more in-depth, multi-marker, analysis of the anti-inflammatory effects of proSAAS overexpression and their potential contribution to therapeutic outcome in the model is needed to validate these preliminary findings.

In conclusion, the present study demonstrates that progression of motor dysfunction can be arrested by proSAAS overexpression in a clinically relevant delayed intervention animal model of PD. Taken together with our previous data pointing to the anti-fibrillation properties of proSAAS in cellular and animal models of PD [2], Alzheimer’s disease [10] and amyotrophic lateral sclerosis [11], this study makes a compelling case for further investigation of potential proSAAS-based therapies for multiple neurodegenerative proteinopathies.

## Supplementary Information

Additional file 1. Supplementary methods.

Additional file 2. **Fig. S1** Representative image of hα-Syn expression in the SNc 8 weeks following AAV-hα-Syn injection in the 4-week-delay experiment. **Fig. S2** Representative image of proSAAS overexpression in the SNc 4 weeks following lenti-proSAAS injection in the 4-week-delay experiment. **Fig. S3** Analysis of Nissl-positive, TH-negative staining. **Fig. S4** Injection of AAV-hα-Syn alone. **Fig. S5** Analysis of Iba-1 immunoreactivity. **Fig. S6** Minimal colocalization of p129-α-Syn and Iba-1 immunoreactivity was apparent in the midbrain in all groups.

## Data Availability

All data generated or analyzed during this study are included in this article (and its supplementary information files).

## Abbreviations

AAV: Adeno-associated virus
α-Syn: Alpha-synuclein
GFP: Green fluorescent protein
hα-Syn: Human alpha-synuclein
Iba-1: Ionized calcium binding adaptor molecule
PD: Parkinson’s disease
SNc: Substantia nigra pars compacta
TH: Tyrosine hydroxylase
