# Supplementary material for: ProSAAS overexpression arrests progression of motor deficits in a rat alpha-synuclein model of Parkinson’s disease
Source: Transl Neurodegener. 2026 Oct 16;15:55. doi: 10.1186/s40035-026-00593-x (PMC13673805; doi:10.1186/s40035-026-00593-x)
Supplement: Supplementary file 1 [file 40035_2026_593_MOESM1_ESM.docx]

**SUPPLEMENTARY METHODS**

Animals

Male Sprague-Dawley rats (Charles River, USA) weighing 250-320 grams at the time of initial surgery were maintained on a 12-12 h light-dark cycle. Food and water were available *ad libitum* throughout the study. All procedures conformed to the National Research Service Guide for the Care and Use of Laboratory Animals, and were approved by UCLA Institutional Animal Care and Use Committee.

Viruses

Adeno-associated viral vectors (AAV 2/1) expressing hα-Syn or GFP, driven by the chicken beta actin promoter and enhanced using a woodchuck hepatitis virus post-transcriptional regulatory element (WPRE), were obtained from the UNC Viral Core at titers of 9.8 and 9.7 x 10^12^ viral genome copies (gc)/ml, respectively. Lentiviruses expressing mouse *Pcsk1n* (proSAAS) or eGFP, driven by the cytomegalovirus (CMV) promoter, were synthesized by GeneCopoiea (Rockville, MD) in the Lv153 backbone at titers of 2.6 x 10^8^ and 2.5 x10^8^ transducing units (TU)/ml, respectively.

Virus Injection

Animals underwent 2 surgeries, carried out under isoflurane anesthesia (5% in oxygen for induction and 2% for maintenance; Zoetis, Kalamazoo, MI). Pre-emptive analgesia was provided by an injection of Carprofen (5 mg/kg s.c.) prior to each surgery and a second dose of Carprofen was administered 24 h after the initial dose. During the first surgery, animals were secured in a stereotaxic frame, with fascia retracted, skull revealed, and 4 burr holes were drilled at the following coordinates above the substantia nigra pars compacta (SNc): AP -4.7 mm, ML +2.1 mm; AP -5.2 mm, ML +1.7 mm; AP -5.6 mm, ML +1.3 mm; AP -5.6 mm, ML +2.4 mm. Animals received 4 unilateral injections into the right SNc of either AAV2/1-hα-Syn or AAV2/1-GFP. Each injection was 1 µl in volume and was delivered into the SNc at a rate of 0.2 µl/min, using the above-mentioned coordinates at a depth of -8.4 mm, -8.6mm, -8.6mm and -7.6mm, respectively, via a 30-gauge 10 µL Hamilton syringe (Hamilton 701SN-30/3'/3) mounted on a motorized injector (Stoelting QSI). After each injection, the syringe remained *in situ* for a further 5 min before withdrawal. The scalp wound was closed with wound-clips and lidocaine injected at the wound margins to provide local anesthesia. During a second surgery, conducted 4 or 6 weeks after the first surgery, animals received injections of lentivirus-mouse proSAAS or lentivirus-eGFP into the same 4 sites in the manner described above through pre-existing burr holes. A separate cohort of animals underwent only the first surgery for injection of AAV2/1-hα-Syn and were euthanized 2, 3, 4, 6 or 8 weeks later.

Motor asymmetry tests

A battery of three tests known to be sensitive to DA depletion, without the need for extensive training, drug administration, or food or water deprivation, was employed, as previously described [1]. Animals were handled and habituated to the behavioral testing procedures for 4 days and 2 baseline scores on each test were subsequently obtained at weekly intervals prior to the initial virus injection, after which weekly testing continued until the 8- or 12-week post-initial injection endpoint. Tests were scored manually without knowledge of group assignment. The *bracing test* probes the ability of the rat to adjust postural balance in response to examiner-imposed lateral movement. The rat was held with only one limb (a forelimb) unrestrained to support its weight and moved laterally at a constant speed across a flat surface for 1 meter in 5 seconds. The number of adjusted steps was recorded twice for both leftward movements with the left forepaw and rightward movements with the right forepaw. Test scores were calculated by dividing the number of steps contralateral to the injected side (leftward movements) by the number of ipsilateral (rightward) steps. The *forelimb placement* test examines sensorimotor/proprioceptive capacity. During the test, the rat’s torso was held with both forelimbs hanging freely and moved slowly sideways toward a vertical flat surface until the vibrissae of one side touched the surface, evoking a characteristic placement of the adjacent forepaw on the surface. Tests of both ipsilateral and contralateral sides were repeated 10 times. The score was calculated as the ratio between the number of successful placements of the contralateral and ipsilateral forelimbs. The *cylinder test* examines spontaneous forelimb utilization during vertical movements. Rats were placed in a clear Plexiglas cylinder (diameter = 20 cm, height = 30 cm) and video-recorded over a 5-min period. During rearing movements, placement of a single paw onto the cylinder wall was recorded as an ipsilateral or contralateral placement relative to the injected side. When both paws were placed on the cylinder simultaneously or in rapid succession, both paws remaining on the cylinder, a score of ‘both’ was recorded. An asymmetry score was calculated as the difference between contralateral (left) and ipsilateral (right) forepaw placements as a percentage of total placements.

Nigro-striatal tyrosine hydroxylase quantitative analyses

*Tissue preparation:* Following the final motor asymmetry test, animals were anesthetized with pentobarbital (100 mg/kg) and transcardially perfused with phosphate buffered saline (PBS) followed by 4% paraformaldehyde in PBS. Brains were harvested, post-fixed in 4% paraformaldehyde overnight at 4^o^C, then cryoprotected in 30% sucrose at 4˚C overnight, and stored at -80˚C. Brains were cut coronally on a cryotome (Leica CM1850) at 40 µm, and sections washed three times in PBS to remove cryoprotectant, incubated in 0.3% H_2_O_2_ for 15 min, and washed again in PBS. Sections were then blocked with 5% normal goat serum/0.5% Triton X-100 in PBS for 2 h at room temperature, followed by overnight incubation at 4˚C with primary rabbit anti-TH antibody (1:1000, Millipore #657012 diluted in blocking solution). The next day, sections were washed 3 times in 3X PBS and once in PBS (10 min/wash). Sections were incubated with secondary antibody (goat anti-rabbit 1:300, Vector Labs #BA-1000) diluted in 2% normal goat serum/0.1% Tween for 2 h. Sections were washed in 0.1% Tween/PBS and then incubated for 1 h in a 0.1% Tween/PBS solution containing an avidin-biotin complex (Vectastain, Vector Labs). After subsequent washing in 0.1% Tween/PBS, the sections were developed using diaminobenzidine (DAB, Sigma) and mounted on glass slides separated by region of interest (striatum or SNc) and left to air dry. Nissl staining was performed 24 h after immunostaining by rehydrating the sections in water followed by incubation in a filtered cresyl violet solution for 10 min, followed by a quick wash, and immersion in 95% ethanol/0.1% glacial acetic acid. Sections were then dehydrated in increasing concentrations of ethanol, soaked in xylene, and mounted with Eukitt mounting medium.

*Nigral unbiased stereology*: Cell counting was performed by an investigator blinded to the virus injection group using Stereo Investigator (MBF Bioscience) coupled with a Leica DM-LB microscope and a Ludl XYZ motorized stage. One in every 8 sections was used to estimate the number of neurons in the SNc in the virus-injected hemisphere and in the contralateral hemisphere using the optical fractionator method. The counting frame was 50 x 50 x 20 µm (length x width x dissector height), and the sampling grid was 150 x 150 µm with a 3 µm guard zone at the top and bottom of the section. The average final section thickness was calculated from the measurements at each counting frame. Following delineation of the SNc under a 5X objective, counting was performed with a 100X oil objective. Cell morphology revealed by Nissl staining was used for classification and identification of different cell types in the SNc using criteria detailed elsewhere [2]. Neurons with a visible nucleus stained for TH immunoreactivity were counted as TH-positive neurons. Neurons stained with cresyl violet with a heavily-stained large single nucleus and lacking TH staining were counted as TH-negative neurons.

*Striatal TH optical density assessment*: Immunostained and mounted striatal sections were scanned using an Aperio ScanScope at 10 µm resolution and the striata ipsilateral and contralateral to the injection analyzed with ImageJ software (version 1.53). Boundaries of the striatum at 3 anterior-posterior levels relative to Bregma – anterior +2.16 mm, middle +1.08 mm, posterior -0.05 mm - were determined by comparing anatomical landmarks in sections with a rat brain atlas [3].

Fluorescence immunocytochemical localization of hα-Syn, p129-α-Syn, proSAAS, Iba-1

Midbrain coronal sections collected as described above, were blocked with 5% donkey serum (Jackson ImmunoLab)/0.3% Triton X-100 in PBS for 2 h at room temperature, with shaking. Sections were then incubated in one of the following primary antibodies overnight at 4˚C: mouse anti-hα-Syn (Life Technologies, #180215) at 1:200 dilution; rabbit anti- proSAAS (anti-PCSK1N; Sigma-Aldrich #HPA064734) 1:750 dilution, rabbit anti-Iba-1 (Wako #019-19741) 1:1000 dilution. Sections were washed 3 times with 3X PBS for 10 min/each and 1 time with PBS and incubated for 2 h at room temperature with one of the following secondary antibodies in 2% donkey serum and 0.1% Tween in PBS: Alexa Fluor 647 donkey anti-mouse at 1:200 dilution (Invitrogen, #A31571); Alexa Fluor 555 donkey anti-rabbit at 1:1000 dilution (Invitrogen, cat# A21436). After washing 3 times with 0.1% Tween/PBS and once with PBS, sections were mounted and cover-slipped prior to visualization under a microscope and image capture. Some sections were alternatively processed for phosphorylated α-Syn imaging. Such sections were washed as described above, followed by immersion in 3% H_2_O_2_ for 15 min, and subsequently washed a further three times in PBS. Sections were then blocked with 5% normal goat serum/0.3% Triton X-100/1% BSA in PBS for 2 h at room temperature, followed by overnight incubation with a primary rabbit anti-phospho129-α-Syn antibody (1:750, Abcam #51253) diluted in the same blocking solution. The next day, sections were washed 3 times in 3X PBS and once in PBS (10 min/wash). Sections were incubated with a secondary antibody (goat anti-rabbit 1:300, Vector Labs #BA-1000) diluted in 2% normal goat serum/0.1% Tween for 2 h. The sections were washed in 0.1% Tween/PBS and then incubated for 1 h in 0.1% Tween/PBS solution containing an avidin-biotin complex (Vectastain, Vector Labs). After subsequent washing in 0.1% Tween/PBS, the sections were developed using diaminobenzidine (DAB, Sigma). Brain sections were mounted on glass slides for microscopic analysis and image capture.

Scoring of Iba-1 staining

Four to 5 evenly spaced (280 µm) coronal sections (40 µm thickness) containing SNc from each animal were rated by two investigators blind to group assignment. Each immunostained brain section was examined under a fluorescent microscope (Olympus BX51) with a 10X objective and scored individually by both examiners. A score between 0 and 5 was assigned to each section based on a comparison of the intensity of fluorescence on the injected versus non-injected side. The score assigned to each animal was an average of the individual sections, which was further averaged across the two raters.

Statistical analyses

Comparisons of motor asymmetry scores collected over multiple weeks were analyzed by mixed model ANOVA of aligned rank-transformed data followed post-hoc Tukey-corrected multiple comparisons where appropriate. Multiple group comparisons of post-mortem data were analyzed by mixed model ANOVA followed by Holm- or Tukey-corrected post-hoc multiple comparisons as appropriate. Single group comparisons were by Students’ t-test. All statistical analyses were performed with custom R code (R Project for Statistical Computing – [http://www.R-](http://www.r-/)[project.org/](http://project.org/)).

Methods citations

1. Phillips JM, Lam HA, Ackerson LC, Maidment NT. Blockade of mGluR5 glutamate receptors in the subthalamic nucleus ameliorates motor asymmetry in an animal model of Parkinson’s disease. Eur J Neurosci. 2006;23:151–60.

2. Nair-Roberts RG, Chatelain-Badie SD, Benson E, White-Cooper H, Bolam JP, Ungless MA. Stereological estimates of dopaminergic, GABAergic and glutamatergic neurons in the ventral tegmental area, substantia nigra and retrorubral field in the rat. Neuroscience. 2008;152:1024–31.

3. Paxinos G, Watson C. The rat brain in stereotaxic coordinates [Internet]. San Diego: Academic Press; 1998. http://www.loc.gov/catdir/description/els033/98122529.html
