# Supplementary material for: ProSAAS overexpression arrests progression of motor deficits in a rat alpha-synuclein model of Parkinson’s disease
Source: Transl Neurodegener. 2026 Oct 16;15:55. doi: 10.1186/s40035-026-00593-x (PMC13673805; doi:10.1186/s40035-026-00593-x)
Supplement: Supplementary file 2 [file 40035_2026_593_MOESM2_ESM.docx]

**SUPPLEMENTAL FIGURES**

**Fig. S1.** Representative image of hα-Syn expression in the SNc 8 weeks following AAV-hα-Syn injection in the 4-week-delay experiment.

**Fig. S2.** Representative image of proSAAS overexpression in the SNc 4 weeks following lenti-proSAAS injection in the 4-week-delay experiment.

**Fig. S3.** **Analysis of Nissl-positive, TH-negative staining. a.** There was no increase in TH-negative neurons in the SNc ipsilateral to the AAV-hα-Syn injection relative to the contralateral side in the 4-week-delay experiment in animals treated with lenti-GFP or lenti-proSAAS. Mixed 2-way ANOVA revealed no main effects of side (*F*_(1,8)_ = 0.01 *p* = 0.9) or treatment (*F*_(1,8)_ = 4.4, *p* = 0.07) and no side x treatment interaction (*F*_(1,8)_ = 0.1, *p* = 0.7). N = 5/group; Error bars +/-SEM. **b.** There was no increase in TH-negative neurons in the SNc ipsilateral to the AAV-hα-Syn injection relative to the contralateral side in the 6-week-delay experiment in animals treated with lenti-GFP or lenti-proSAAS. There was, however, an unexpected statistically significant bilateral elevation in TH-negative neurons in proSAAS-treated animals relative to both hα-Syn+GFP and GFP+GFP groups. The reason for this is unclear but one plausible, albeit speculative, explanation is that proSAAS offers protection from a non-specific, bilateral toxicity of unilateral viral injection to non-dopaminergic cells. Mixed 2-way ANOVA showed a main effect of treatment (*F*_(2,17)_ = 7.3, *p* < 0.01), no effect of side (*F*_(1,17)_ = 0.5, *p* = 0.5) and no treatment x side interaction (*F*_(2,17)_ = 1.5, *p* = 0.3). Tukey-corrected post-hoc comparisons showed significant differences between hα-Syn+proSAAS and both hα-Syn+GFP (****p*<0.001) and GFP+GFP (***p*<0.01) groups. N = 6-7/group; Error bars, ±SEM. **c.** There was no evidence of an increase in the number of TH-negative neurons in the SNc on the injected side relative to the contralateral side across time in animals injected with AAV-hα-Syn alone. Two-way mixed ANOVA showed no significant effect of time (*F*_(4,10)_ = 0.8, *p* = 0.6) or side (*F*_(1,10)_ = 0.2, *p* = 0.7). While the time x side interaction approached significance (*F*_(4,10)_ = 3.3, *p* = 0.06), no clear pattern was apparent. N = 3/group; Error bars, ±SEM.

**Figure S4. Injection of AAV-hα-Syn alone. a**. p129-α-Syn-positive cells (EP1536Y, Abcam #51253) were apparent as early as 2 weeks post-injection on the injected side only, their number gradually declining from 3 to 8 weeks: one-way ANOVA *F*_(4,10)_ = 5.32; *p* < 0.05, N = 3/group; Error bars +/- SEM. **b**. Asymmetry in TH+ cell counts in the SNc of animals injected with AAV-hα-Syn alone, measured stereologically, was similarly apparent as early as 2 weeks post-injection, progressing in amplitude thereafter: two-way mixed ANOVA; main effects of time (*F*_(4,10)_ = 3.5, *p* < 0.05), and side of brain (*F*_(1,10)_ = 407.4, *p* < 0.001), and time x side interaction (*F*_(4,10)_ = 5.7, *p* < 0.05). N = 3/group; Error bars +/- SEM. **c**. Asymmetry in striatal TH optical density was similarly apparent at 2 weeks and progressed thereafter. Three-way mixed ANOVA demonstrated significant main effects of time (*F*_(4,10)_ = 11.1, *p* < 0.01), side of brain (*F*_(1,10)_ = 185.5, *p* < 0.001) and rostro-caudal region (*F*_(2,20)_ = 122.6, *p* < 0.001), as well as a 3-way interaction (*F*_(8,20)_ = 2.5, *p* < 0.05). Follow-up one-way ANOVA demonstrated a significant effect of time only on the side of injection (*F*_(4,10)_ = 18.5, *p* < 0.001). N = 3/group; Error bars, ±SEM.

**Fig. S5.** **Analysis of Iba-1 immunoreactivity. a.** Example sections from the 6-week delay experiment showing the scoring range for Iba-1 immunoreactivity in the midbrain. **b.** There was no significant difference in Iba-1 scores at 6-8 weeks in animals injected with AAV- hα-Syn alone, and scores at 8 weeks in animals injected with AAV-hα-Syn + lenti-GFP or AAV-hα-Syn + lenti-proSAAS from the 4-week-delay experiment. One-way ANOVA of aligned ranked transformed data showed no main effect of treatment (*F*_(2,13)_ = 1.5, *p* = 0.3). N = 5-6/group; Error bars +/-SEM. **c.** A comparison of Iba-1 immunoreactivity at 12 weeks in the 6-week-delay experiment revealed significantly lower levels in the hα-Syn+proSAAS group compared to both hα-Syn+GFP and GFP+GFP groups. One-way ANOVA of aligned ranked transformed data showed a main effect of treatment (*F*_(2,17)_ = 13.2, *p*<0.001). Tukey-corrected post-hoc pairwise comparisons are indicated *p<0.05, ***p<0.001. N = 6-7/group; Error bars, ±SEM.

**Fig. S6.** Minimal colocalization of p129-α-Syn and Iba-1 immunoreactivity was apparent in the midbrain in all groups; the example shown is from an animal injected with AAV-hα-Syn + lenti-proSAAS in the 4-week delay experiment taken at 8 weeks.
